# Methane Production From Different Parts of Corn Stover via a Simple Co-culture of an Anaerobic Fungus and Methanogen

**DOI:** 10.3389/fbioe.2020.00314

**Published:** 2020-04-30

**Authors:** Yuqi Li, Zhesheng Hou, Qicheng Shi, Yanfen Cheng, Weiyun Zhu

**Affiliations:** ^1^Laboratory of Gastrointestinal Microbiology, National Center for International Research on Animal Gut Nutrition, Nanjing Agricultural University, Nanjing, China; ^2^Joint International Research Laboratory of Animal Health and Food Safety, Nanjing Agriculture University, Nanjing, China; ^3^College of Mechanical and Electrical Engineering, Jilin Institute of Chemical Technology, Jilin, China

**Keywords:** anaerobic fungus, methanogen, corn stover, leaf blade, stem pith

## Abstract

To determine ways to improve the utilization of corn stover, this study investigated methane production from different parts of corn stover using a simple co-culture of an anaerobic fungus (*Pecoramyces* species) and methanogen (*Methanobrevibacter* species). The simple co-culture was incubated with the stem pith, leaf blade, or stem bark of corn stover (as substrates) at 39°C for 72 h. The results showed that the stem bark had the lowest (*P* < 0.05) digestibility (38.0 ± 1.36%) and neutral detergent solubles, that is, cell solubles (31.6 ± 0.45%), and the highest (*P* < 0.05) lignin content (4.8 ± 0.56%). The leaf blade had a significantly higher methane conversion rate (56.6 ± 0.76 mL/g digested substrate) than the stem pith (49.2 ± 1.60 mL/g digested substrate), even though they showed similar levels of methane production (42.4 ± 1.0 mL and 40.9 ± 1.35 mL, respectively). Both the leaf blade and stem pith of corn stover have the potential to produce methane in a simple co-culture of an anaerobic fungus and methanogen.

## Introduction

The rational exploitation and utilization of energy are vital for sustainable social development. The increasing depletion of fossil fuels is one of the biggest challenges for the future development of the economy and society. The production of non-petroleum sources of energy has attracted increased attention in several countries. Today, dedicated energy crops, such as maize, sorghum, and wheat, are widely used to produce methane (Riva et al., 2014). However, an increasing energy demand has led to the need to consume large amounts of such energy crops to produce methane. This makes the cost of methane production very high, and leads to an inevitable conflict between the use of limited supplies of energy crops for both food/feed and energy production (Croce et al., 2016).

The use of corn stover, instead of energy crops, to produce methane is one way to address this challenge. Corn stover stores half of the organic matter of an entire crop and has a reasonably high nutritional value (Li et al., 2017). However, cellulose, which is complexed with lignin in crop residues, is highly resistant to anaerobic fermentation and thus to methane production (Berchem et al., 2017; Wu and Tian, 2018). Although there are many limitations in the production of methane from corn stover, it is still considered a good potential substrate for anaerobic fermentation. Furthermore, the production of methane from corn stover is greater than that produced from other crop straws (Croce et al., 2016). In recent years, corn stover has been used to produce methane in anaerobic digestors or in batch cultures (Haag et al., 2015).

Methane production from corn stover depends on its chemical composition (Fernando et al., 2006). At present, some physical and chemical pretreatment methods can improve the degradation of straw and thus improve the efficiency of methane production. Steam explosion, microwave, and other heat treatment methods can dissolve lignin, but they also produce toxic substances, which can inhibit anaerobic fermentation (Sapci et al., 2013; Theuretzbachera et al., 2015). Although cold treatment methods, such as extrusion and comminution, do not produce harmful substances, they do not remove lignin from the fiber structure, which leads to poor methane production (Hjorth et al., 2011).

Other processes, such as acid and alkali pretreatment, may also be applied separately before anaerobic fermentation. The application of acid for pretreatment is great extent restricted by high acid and energy consumption, equipment corrosion, and obligation for acid recovery (Ibrahim, 2012). Moreover, alkaline treatment can even produce secondary products, thereby reducing the production of methane during fermentation (Croce et al., 2016). When combined with some physical and chemical methods to simultaneously pretreat straw, the above listed drawbacks could be alleviated, and methane production increased. However, such pretreatment itself violates the original intention of producing new energy sustainably, owing to the considerable consumption of energy. In addition, the application of such treatments can be expensive.

Anaerobic fungi (AF), a constituent of the natural microbial communities found in the rumen of herbivores, are known for their fiber-degrading ability. Unlike fiber-degrading bacteria, AF have unique rhizoid systems that can colonize and degrade the plant cell wall for effective degradation (Gruninger et al., 2014; Solomon et al., 2016). Anaerobic fungi use many carbohydrate-degrading enzymes and their unique rhizoid system to physically destroy the ultrastructure of the plant cell wall to degrade lignocellulose. This action is considered to increase the surface area for bacterial colonization and further enzymatic digestion (Gruninger et al., 2018). The use of AF could preclude the requirement for pretreatment in methane production.

Compared with industrial preparations, AF are natural, harmless, efficient, and convenient and can be easily obtained. Methanogens can be found toward the end of the metabolic process in the rumen, which can utilize the metabolic products of AF and fibrolytic bacteria to produce methane (Jin et al., 2017). Many studies using co-cultured AF and methanogens have shown that co-cultured methanogens could not only use fungal metabolites to produce methane, but also shift the fungal metabolic pathway to confer significantly higher levels of fiber-degrading ability to the AF (Joblin et al., 1990). Thus, co-cultures of AF and methanogens have the potential for use in the degradation of lignocellulosic substrates for methane production (Theodorou et al., 1996; Bootten et al., 2011; Shi et al., 2019).

Zhou et al. (2015) reported that the structural components and nutrient utilization rates of different parts of the same straw were significantly different. Zhao et al. (2011) demonstrated that the content and structure of cellulose and hemicellulose in the stem bark (SB), leaf blade (LB), and stem pith (SP) of corn stover also differed. In order to improve the utilization of corn stover, this study investigated the methane production from different parts of corn stover using a simple co-culture of an anaerobic fungus and methanogen.

## Materials and Methods

### Co-culture of Anaerobic Fungus and Methanogen

The co-culture of an anaerobic fungus and methanogen used in the present study was isolated from a goat (Jin et al., 2011). The fungus was identified as *Pecoramyces* species (Li et al., 2017), and the methanogen was identified as *Methanobrevibacter* species (Jin et al., 2011). The anaerobic fungus was identified to the genus level through the use of traditional morphological identification and molecular phylogenetic analyses. Morphological identification included rhizoid, mycelium, and flagella identification and nuclear staining with DAPI (4′,6-diamidino-2-phenylindole). Molecular phylogenetic analyses were based on the amplification and sequencing of the genes encoding the 28S rRNA (*LSU*) and ITS (internal transcribed spacer) sequences with AF-LSU primers (Li et al., 2019). The 16S rRNA genes amplified from the total DNA extracted from the cultures were used for gene sequencing to evaluate the specific type of methanogen, using the Met86F and Met1340R primers, according to the methods of Jin et al. (2011).

The co-culture was maintained in liquid media (Davies et al., 1993) with rice straw as a substrate and transferred every 3 days. Briefly, this method entailed the transfer of 1 mL of co-cultured anaerobic fungus and methanogen solution into a roll tube with 9 mL of fresh medium, according to the methods of Cheng et al. (2009). Each 1,000 mL of medium contained 150 mL of buffer solution A, 150 mL of buffer solution B, 550 mL of basal medium, (2.5 g yeast extract, 100 g tryptone, and 6 g NaHCO_3_), 150 mL of cell-free rumen fluid (centrifuged at 16,000*g* for 20 min at 4°C, with the supernatant decanted and stored at −20°C), 1 g of L-cysteine hydrochloride, and 1 mL of 0.1% (wt/vol) resazurin. Buffer solution A contained 0.3 g of K_2_HPO_4_ per 100 mL, and buffer solution B contained 0.3 g of KH_2_PO_4_, 0.6 g of NaCl, 0.6 g of (NH_4_)_2_SO_4_, 0.06 g of MgSO_4_ ⋅ 7H_2_O, and 0.06 g of CaCl_2_ ⋅ 2H_2_O per 100 mL. This medium was sterilized by autoclaving at 115°C for 20 min.

Penicillin–streptomycin solution was added to the medium to inhibit bacterial growth. The final concentrations of penicillin and streptomycin were 1,915 and 2,031 U mL^–1^, respectively. Antibiotic solution was sterilized by using a filtration membrane (size 0.22 μm, SCAA-102; ANPEL, Shanghai, China). At the end of the fermentation process, an aliquot of 10 mL of the supernatant from each bottle was transferred to new media, to which chloramphenicol was added to determine whether the medium became clear after 3 days of incubation. If the medium became clear, this indicated the presence of methanogens in the fermentation medium, and if the medium remained turbid, this indicated the presence of bacteria.

### Different Parts of Corn Stover

The corn stover was collected, air dried, and separated into the LB, SP, and SB. The three parts were separately oven dried at 65°C and ground to be passed through a sieve (∼1 mm) for further use as substrates.

### Experimental Design and Sample Collection

The experiment comprised three groups, each containing 1 g of SP, LB, or SB as substrates. Each group had four replicates. The media (90 mL) was pre-warmed at 39°C and inoculated with 10 mL of 3-day-old anaerobic fungus and methanogen co-culture. Based on the established growth characteristics of the anaerobic fungus (Li et al., 2017), the co-culture of the anaerobic fungus and methanogen was conducted under strict anaerobic conditions under a headspace of 100% CO_2_ at 39°C in a butyl rubber-stoppered 180 mL serum bottle containing 90 mL of media without shaking for 72 h. A blank group without inoculation was prepared for gas and analyte correction.

Before the fermentation process, 2 mL of the supernatants was collected from the SP, LB, and SB vessels and used to measure the concentrations of reducing sugars, glucose, and xylose. Gas production and methane production were measured every 6 h (at 0, 6, 12, 18, 24, 30, 36, 42, 48, 54, 60, 66, and 72 h). At the end of fermentation, the pH was immediately measured, and the supernatant was collected for the analysis of fermentation end products, fiber-degrading enzyme activities, and reducing sugars. The remaining substrates were collected for the analysis of the digestibility of dry matter (DM), neutral detergent fiber (NDF), acid detergent fiber (ADF), cellulose, and hemicellulose.

### Analysis of Chemical Composition of Different Parts of Corn Stover

The contents of NDF, ADF, and lignin of the LB, SP, and SB were determined according to the methods of Van Soest et al. (1991). The cell solubles or the soluble solutes in the neutral detergent solution (NDS), cellulose, and hemicellulose were calculated according to the methods of Niu et al. (2018). In addition, NDF and ADF were determined using a fiber analyzer (Ankom A200i; Ankom Technology, Macedon, NY, United States). First, the samples were treated with NDS; the dissolved part was NDS, and the residue was NDF. The NDF was further treated with acid detergent solution to dissolve the hemicellulose and obtain the ADF. The ADF thus obtained was digested with 72% sulfuric acid, which dissolves cellulose, and the residue comprised a mixture of lignin and silicate. This residue was ashed, which removes the lignin, thereby facilitating the determination of silicate. The digestibility of DM, NDF, ADF, cellulose, and hemicellulose was calculated according to the methods of Li et al. (2016).

Each 1,000 mL of NDS contained 30 g of sodium dodecyl sulfate, 18.6 g of disodium ethylenediamine tetraacetic acid (Na_2_EDTA), 6.8 g of Na_2_B_4_O_7_, 4.6 g of Na_2_HPO_4_, and 10 mL of C_6_H_14_O_4_. Each 1,000 mL of acid detergent solution contained 20 g of cetrimonium bromide dissolved in 1,000 mL of 0.5 mol/L sulfuric acid solution.

### Measurement of Gas and Methane Production

Gas production was determined using a pressure transducer, according to the methods of Theodorou et al. (1994). The pressure transducer determined the levels of gas production during fermentation at the top surface of the serum bottles with a capacity of 180 mL. Gas production was recorded every 6 h, and the gas was then released to bring the air pressure within the bottle to 0, to facilitate the determination of gas production at the next time point. The gas production volumes at each time point were added to determine the cumulative gas production. By recording the gas production at intermittent time points, a complete growth curve of the co-culture of the anaerobic fungus and methanogen was obtained. After each time point at which gas production was determined, 5 mL of gas was collected in an air bag to determine the methane concentration in the gas.

Methane content in the gas was determined by gas chromatography (Agilent 7890B; Agilent, Palo Alto, CA, United States), according to the methods of Hu et al. (2006). The conditions used were as follows: column temperature of 80°C; vaporization chamber temperature of 100°C; H_2_ ion flame detector, with a detection temperature of 120°C; and carrier gas (N_2_) pressure of 0.05 MPa; air pressure of 0.05 MPa; and H_2_ pressure of 0.05 MPa. The volume of methane was calculated according to the method of Li et al. (2018).

### Analysis of Fermentation End Products

The reducing sugars were analyzed using the 3,5-dinitrosalicylic acid (DNS) method (Lowe et al., 1987b). The ratio of the sample to DNS reagent was 1:2, and absorbance was read at 640 nm. The concentrations of glucose, xylose, formate, acetate, and lactate were determined according to the protocol described by Shi et al. (2019). Glucose was determined using the glucose oxidase method, xylose was measured using the phloroglucinol color-developing method, and lactate was determined by the NAD^+^ transformation method. These analytes were all determined using the applicable assay kits (Nanjing Jiancheng Bioengineering Institute, Nanjing, China).

Formate was measured with the Formate Assay Kit (Sigma, Santa Clara, CA, United States). Acetate was measured by gas chromatography (Daojin GC- 2014AFsc Instrument; Shimadzu, Kyoto, Japan) using a capillary column. An aliquot of 0.2 mL of crotonate metaphosphate solution (0.25 g/mL) was mixed with 1 mL of supernatant from the fermentation medium and centrifuged at 16,000*g* for 10 min. The supernatant was then analyzed by gas chromatography with a column temperature of 130°C; vaporization temperature of 180°C; H_2_ ion flame detector and detection temperature of 180°C; N_2_ carrier gas at a pressure of 60 kPa; H_2_ pressure of 50 kPa; and O_2_ pressure of 50 kPa. Ethanol was determined by gas chromatography (TRACE GC Ultra; Thermo Fisher, Waltham, Massachusetts, United States) using the method described by Edgardo et al. (2008).

### Analysis of Fiber-Degrading Enzyme Activity

The activities of carboxymethyl cellulase (CMCase), a cellulose-degrading enzyme, and xylanase, a hemicellulose-degrading enzyme, were measured according to the method of Lowe et al. (1987a). The fermentation supernatant was used to measure enzyme activities. To an appropriate volume of the supernatant solution (preheated at 50°C), either 1 mL of xylan solution or 10 mg of carboxymethylcellulose sodium in 0.1 mol/L citric acid disodium hydrogen phosphate buffer solution was added. After 30 min of reaction at 50°C, DNS reagent was added, and the mixture was then boiled for 10 min, and the absorbance was read at 640 nm. The ratio of the sample to DNS reagent was 1:2. One unit of CMCase activity was defined as 1 μmol of glucose released per mL of supernatant per minute (U mL^–1^ min^–1^). One unit of xylanase enzyme activity was defined as 1 μmol of xylose released per mL of supernatant per minute (U mL^–1^ min^–1^).

### Statistical Analysis

Statistical analysis was performed using the SPSS 20.0 software (IBM SPSS Statistics, version 20.0; IBM Corp, Armonk, NY, United States) with one-way analysis of variance at a confidence interval of 95%. Duncan new multiple-range test was then used to compare the differences among the three groups. Data were presented as the mean ± standard error of the mean.

## Results

### Chemical Composition and Digestibility of the SB, LB, and SP of Corn Stover

The results are presented in Table 1. The SB had the lowest (*P* < 0.05) NDS content (31.6 ± 0.45%) and highest (*P* < 0.05) cellulose (40.9 ± 0.30%) and lignin (4.8 ± 0.56%) contents. The LB had the lowest (*P* < 0.05) cellulose (26.5 ± 0.09%) and lignin contents (2.4 ± 0.07%), and highest (*P* < 0.05) hemicellulose content (27.4 ± 0.55%). The SP had the highest (*P* < 0.05) NDS content (45.4 ± 0.55%).

**TABLE 1:** Chemical composition of the stem bark, leaf blade, and stem pith of corn stover.

**Items**	**Corn stover parts**			**SEM**	***P***
	**SB**	**LB**	**SP**		
NDS (%)	31.6^c^	42.8^b^	45.4^a^	0.73	<0.001
NDF (%)	68.4^a^	57.2^b^	54.6^c^	0.73	<0.001
ADF (%)	46.6^a^	29.8^c^	31.4^b^	0.48	<0.001
Cellulose (%)	40.9^a^	26.5^c^	28.0^b^	0.42	<0.001
Hemicellulose (%)	21.8^b^	27.4^a^	23.3^b^	0.90	0.002
Lignin (%)	4.8^a^	2.4^b^	2.6^b^	0.62	0.013

**Values in the same row with different superscript letters are significantly different (P < 0.05). SB, stem bark; LB, leaf blade; SP, stem pith; NDS, neutral detergent soluble solute; NDF, neutral detergent fiber; ADF, acid detergent fiber; SEM, standard error of the mean (n = 4).**

**FIGURE 1:**
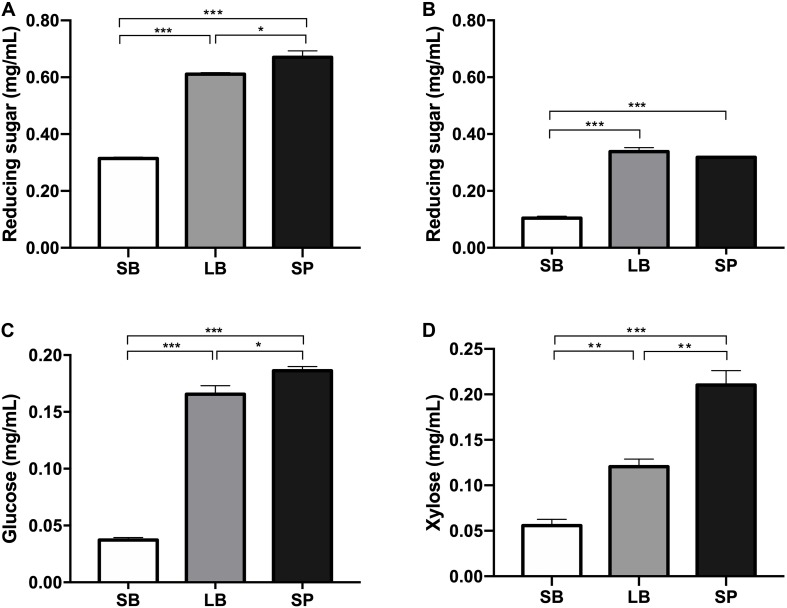
Concentrations of reducing sugar before **(A)** and after **(B)** fermentation and of glucose **(C)** and xylose **(D)** in the supernatant, following incubation using the stem bark (SB), leaf blade (LB), and stem pith (SP) of corn stover as substrates. The error bars represent the standard error of the mean (*n* = 4). *P < 0.05, **P < 0.01, and ***P < 0.001.

The degradation values of the SB, LB, and SP of corn stover in the co-culture are shown in Table 2. The DM digestibility (DMD) of the SP was the highest (82.0 ± 1.95%, *P* < 0.05), followed by those of the LB (74.8 ± 1.95%) and SB (38.0 ± 1.36%). The hemicellulose digestibility of the SP was highest (78.6 ± 1.64%), followed by those of the LB (77.4 ± 0.95%) and SB (25.7 ± 0.75%). As shown in Figure 1, the concentrations of reducing sugars, glucose, and xylose in the SP were the highest (*P* < 0.05), followed by those in the LB and SB.

**TABLE 2:** Degradation of the stem bark, leaf blade, and stem pith of corn stover in a co-culture of an anaerobic fungus and methanogen.

**Items**	**Corn stover parts**			**SEM**	***P***
	**SB**	**LB**	**SP**		
DMD (%)	38.0^c^	74.8^b^	82.0^a^	2.51	<0.001
NDFD (%)	32.7^c^	63.3^b^	79.5^a^	0.17	<0.001
ADFD (%)	36.0^c^	50.3^b^	80.2^a^	0.12	<0.001
NDSD (%)	62.4^c^	81.4^b^	86.9^a^	0.07	<0.001
HD (%)	25.7^b^	77.4^a^	78.6^a^	0.30	0.002
CD (%)	24.2^c^	60.1^b^	75.0^a^	0.75	< 0.001

**Values in the same row with different superscript letters are significantly different (P < 0.05). SB, stem bark; LB, leaf blade; SP, stem pith; DMD, digestibility of dry matter; NDFD, digestibility of neutral detergent fiber; ADFD, digestibility of acid detergent fiber; NDSD, digestibility of neutral detergent solubles; HD, digestibility of hemicellulose; CD, digestibility of cellulose; SEM, standard error of the mean (n = 4).**

### Gas and Methane Production From the SB, LB, and SP of Corn Stover

**FIGURE 2:**
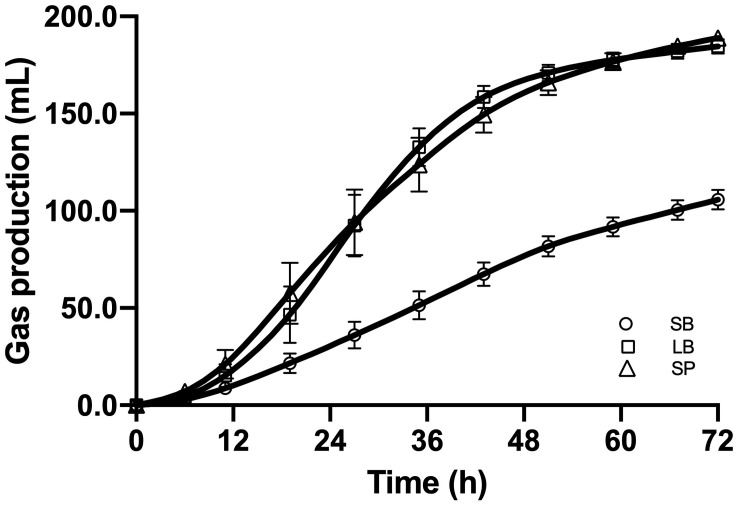
Cumulative gas production from the stem bark (SB), leaf blade (LB), and stem pith (SP) of corn stover using a co-culture of anaerobic fungus and methanogen. The error bars represent the standard error of the mean (*n* = 4).

**FIGURE 3:**
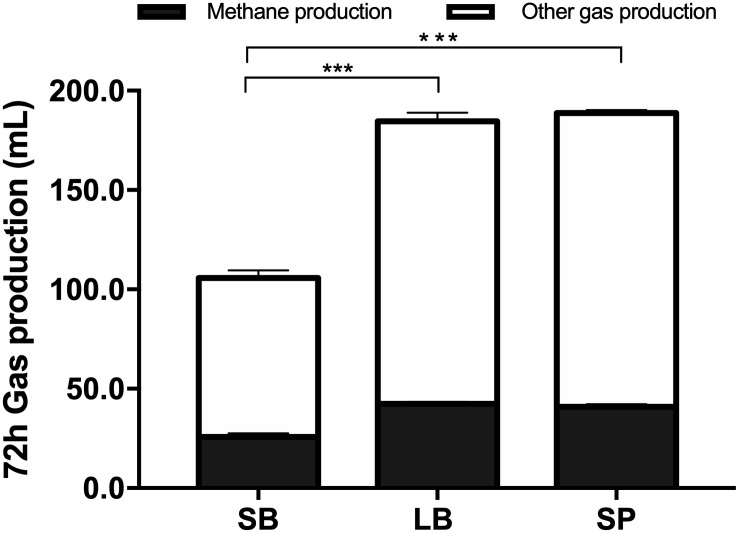
Total gas and methane production from the stem bark (SB), leaf blade (LB), and stem pith (SP) of corn stover using a co-culture of anaerobic fungus and methanogen. The error bars represent the standard error of the mean (*n* = 4). ***P < 0.001.

Figure 2 shows the cumulative gas production, over 72 h of fermentation, from the SB, LB, and SP of corn stover using the co-culture. The curves of methane production were similar to those of overall gas production. At the end of fermentation, the total gas production values of the LB (184.6 ± 3.74 mL) and SP (188.8 ± 2.60 mL) were significantly higher than those of the SB (105.8 ± 5.06 mL, *P* < 0.05), and no significant difference was observed between those of the LB and SP (*P* > 0.05) (Figure 3). Total methane production values from the LB (42.4 ± 0.99 mL) and SP (40.9 ± 1.35 mL) were significantly higher than those from the SB (25.8 ± 1.85 mL, *P* < 0.05), and no significant difference was observed between those from the LB and SP (*P* > 0.05) (Figure 3). The LB and SP showed similar levels of methane production, but the DMD of the LB was significantly lower than that of the SP (*P* < 0.05). This indicated that the methane conversion rate of the LB (56.6 ± 0.76 mL/g digested substrate) was significantly higher than that of the SP (49.2 ± 1.60 mL/g digested substrate) (*P* < 0.05).

### Fiber-Degrading Enzyme Activity and Fermentation Metabolites Following Incubation of the SB, LB, and SP of Corn Stover

As shown in Table 3, the activity of CMCase in the LB group was significantly higher (*P* < 0.05) than that in the SP and SB groups. Xylanase activity in the SB group was significantly lower (*P* < 0.05) than that in the LB and SP groups, and no significant difference (*P* > 0.05) was observed between the values in the LB and SP groups.

**TABLE 3:** Activities of fiber-degrading enzymes of an anaerobic fungus following incubation using the stem bark, leaf blade, and stem pith of corn stover as substrates.

**Items**	**Corn stover parts**			**SEM**	***P***
	**SB**	**LB**	**SP**		
CMCase (U mL^–1^ min^–1^)	0.37^c^	0.50^a^	0.45^b^	0.01	<0.001
Xylanase (U mL^–1^ min^–1^)	9.51^b^	14.53^a^	13.80^a^	0.71	<0.001

**Values in the same row with different superscript letters are significantly different (P < 0.05). SB, stem bark; LB, leaf blade; SP, stem pith; SEM, standard error of the mean (n = 4); CMCase, carboxymethyl cellulase.**

**FIGURE 4:**
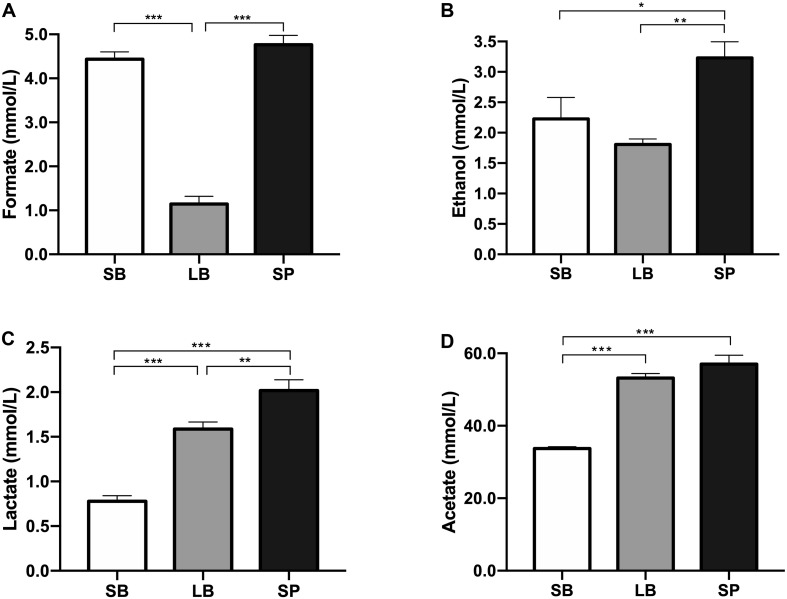
Concentrations of formate **(A)**, ethanol **(B)**, lactate **(C)**, and acetate **(D)** in the supernatant of a co-culture of anaerobic fungus and methanogen, following incubation using the stem bark (SB), leaf blade (LB), and stem pith (SP) of corn stover as substrates. The error bars represent the standard error of the mean (*n* = 4). *P < 0.05, **P < 0.01, and ***P < 0.001.

The pH values showed significant differences among the three groups (*P* < 0.05). The SB had the highest pH value (6.6 ± 0.01), which was significantly higher than those of the LB (6.4 ± 0.02) and SP (6.3 ± 0.01) (*P* < 0.05). The pH value of the LB was also significantly higher than that of the SP (*P* < 0.05). The concentrations of water-soluble metabolites in the supernatant of the co-culture of the anaerobic fungus and methanogen when incubated with SB, LB, and SP as substrates are shown in Figure 4. Acetate was the dominant metabolite in the supernatant, followed by formate, ethanol, and lactate. The concentrations of formate, ethanol, lactate, and acetate all showed significant differences among the three groups (*P* < 0.05).

## Discussion

The DMD of substrates reflects their utilization (Hao, 2011). In the present study, the DMD of the SP, LB, and SB of corn stover showed significant differences (*P* < 0.05). The DMD of the SB was significantly lower than that of the SP and LB, and the DMD of the LB was significantly lower than that of the SP (*P* < 0.05). These findings might be due to the differences in cell-soluble and lignin contents among the three parts. According to Ma et al. (2018), NDS mainly contains protein, fat, starch, and sugar, all of which can be easily degraded by microorganisms (Teunissen et al., 1992).

In the present study, NDS in the SP was the highest, and that in the SB was the lowest. In plant fibers, lignin and hemicellulose are assembled into a complex supramolecular network, which coats the cellulose fibrils. This complex network reduces the digestibility of plant fiber and is the major constraint in the sustainable production of biofuels (Gruber, 2009; Silveira et al., 2015). In the present study, the digestibility of hemicellulose and cellulose in the LB and SP was significantly higher than that in the SB, which might be due to the significantly higher lignin content in the SB compared with that in the LB and SP.

Sun et al. (2005) investigated the attachment on and fermentation profiles of substrates with different lignin contents exposed to ruminal AF. They found that the growth of AF was decreased with increasing lignin content in the substrates. Lignin is a complex compound composed of phenylpropane. It is the most difficult part of the plant cell wall to be degraded. Lignin and polysaccharide are linked by hydroxycinnamic acid, which forms an ester bond and ether linkage with carbonyl and phenolic groups of hydroxycinnamic acid. Although AF can degrade arabinoxylan and release hydroxycinnamic acid, they can neither break the ether bond between lignin and hydroxycinnamic acid, nor degrade lignin (Borneman et al., 1990). Therefore, AF can degrade plant tissues with a relatively low lignin content to a greater extent and at a higher rate.

Methane production by the SB was the lowest in this study, which might be due to the low DMD of the SB, resulting in less substrates (H_2_/CO_2_/formate) for the co-cultured methanogens. Zhu et al. (2001) determined the total gas and methane production in their study of the degradation of straw by an anaerobic fungus. They found that the DMD was positively correlated with total gas production and methane production. Jin (2009) also reported that methane production was positively correlated with the DMD of substrates following the co-culture of an anaerobic fungus and methanogen. The process of anaerobic fungal growth and the utilization of crude fiber can be divided into two stages: in the first phase, the polysaccharide hydrolytic enzyme secreted by the anaerobic fungus hydrolyzes the crude fiber into fermentable sugars (mainly glucose and xylose); in the second stage, the anaerobic fungus absorbs or transports these fermentable sugars into the cell and finally metabolizes them into H_2_, CO_2_, formate, acetate, lactate, and ethanol (Solomon et al., 2016). In this study, each part of corn stover was first degraded into a large number of fermentable carbon sources, and then these carbon sources generated H_2_, CO_2_, and soluble metabolites in the cytosol and hydrogenosome of anaerobic fungus. At the end of the fermentation, the measured amount of H_2_ accumulation was very low, accounting for only 2% of the total gas production, indicating that most of the H_2_ produced was used by methanogens, H_2_ reduces CO_2_ to generate methane.

Li (2017) reported that during co-culture methane production is accelerated during the period from 32 to 64 h, and methanogens can utilize both the H_2_ and formate produced by the anaerobic fungus. In our current study, the LB fraction with the highest methane conversion efficiency had the lowest formate concentration in the medium. In addition, gas production from the LB fraction was highest at 30–64 h of fermentation. These results suggest that a large amount of formate produced by the anaerobic fungus was utilized by methanogens during this fermentation stage, which is consistent with previous results.

The SP had the highest DMD in the present study, which might be explained by the higher content of NDS and lower contents of cellulose and lignin, as discussed above. It is interesting that the LB had a significantly lower DMD than the SP but showed similar levels of gas and methane production. Thus, the methane conversion efficiency of the LB was higher than that of the SP, which might be attributed to higher levels of hemicellulose degradation in the LB compared with that in the SP. Without considering economic performance, the methane conversion efficiency of the co-culture pretreatment strategy used in the present study was lower than that of the physical and chemical pretreatment mentioned in the introduction. Whether sulfuric acid, hydrochloric acid, sodium hydroxide, extrusion, or steam explosion pretreatment is used, the conversion efficiency of methane is consistently higher than 100 mL/g, which is more than twofold the conversion efficiency of the pretreatment strategy used in the present study. However, the pretreatment of sulfuric acid, hydrochloric acid, and sodium hydroxide took 9 days (7 days of acid treatment and 2 days of drying), and the following anaerobic digestion was up to 35 days, which indicated that the whole procedure was 44 days (Song et al., 2014; Croce et al., 2016). Meanwhile, the present strategy provided in this study took only 3 days for the whole procedure, which is much more efficient than the above discussed pretreatments.

Compared with the NDS, hemicellulose and cellulose in plants need to be gradually degraded into usable sugars by plant cell wall-degrading enzymes secreted by the anaerobic fungus (Mountfort and Asher, 1985; Haitjema et al., 2014). These usable sugars are then metabolized by the anaerobic fungus into H_2_, CO_2_, formate, acetate, ethanol, and succinic acid (Li et al., 2016). Methanogens use H_2_, reduce the partial pressure of H_2_ in the system, remove the inhibition of hydrogenase, and thus catalyze more NAD(P)H to H_2_, which results in increased methane production (Bernalier et al., 1991). The formation of lactate and ethanol requires the participation of NAD(P)H; thus, the production of lactate and ethanol should be inhibited. In this study, the concentrations of ethanol and lactate in the LB group were significantly lower than those in the SP group (*P* < 0.05).

Solomon et al. (2016) showed that hemicellulase expression is more easily regulated than cellulolytic enzymes in AF. Recent studies have also found that under the same conditions the activity of xylanase produced by AF in the rumen is six times that of cellulase (Li et al., 2017) and four times more than that of the xylanase produced in some industrial fermentations (Lee et al., 1999). Yu (2017) studied the activity of plant cell wall–degrading enzymes in 12 strains of an anaerobic fungus and found that the xylanase activity in *Piromyces* species CN6 was significantly higher than that of cellulase. Jin (2009) studied the activities of several enzymes after 96 h of fermentation of rice straw by AF and found that xylanase activity of the AF was much higher than that of cellulase. The results of our current study also reflect this situation, in which xylanase activity was much higher than that of cellulase.

At the end of fermentation, the difference in pH values was due to the differences in the concentrations of metabolites. The accumulation of metabolites (acetate, lactate, and formate) can result in a reduction of the pH. Formate, lactate, acetate, and ethanol are reportedly the main water-soluble metabolites of AF (Boxma et al., 2004; Li et al., 2016). Ethanol and lactate are the end products of cytoplasmic metabolism by AF. Compared with the SP, the low concentrations of ethanol and lactate in the LB indicate that cytoplasmic metabolism by the anaerobic fungus was reduced, and more metabolites entered the hydrogenosome to generate H_2_ and acetate, which promoted the co-cultured methanogen to produce methane, as discussed above. When co-cultured with methanogen, the formate in the supernatant might be utilized to produce methane when H_2_ is limited (Jin, 2012; Wei et al., 2016).

In the present study, significantly lower levels of formate were observed in the LB group, which implied that the formate was used by the co-cultured methanogen to produce methane, resulting in higher levels of methane in this group. Previous studies have shown that the co-culture of an anaerobic fungus and methanogen could degrade lignocellulosic substrates and produce methane with very limited amounts of formate in the supernatant within 3 days (Kim et al., 2011; Tapio et al., 2017). In the present study, the concentrations of formate were all greater than 1 mmol/L, which implies that the co-culture needed more time to utilize formate to produce methane. Moreover, a large amount of acetate was accumulated in the culture. If acetate-utilizing methanogens could be added to the culture, much higher levels of methane could be produced.

The SB, LB, and SP showed significantly different chemical compositions, which resulted in different levels of digestibility and methane production. The LB and SP of corn stover had higher levels of digestibility and methane production, as they had higher levels of NDS and hemicellulose, which could be easily degraded by the anaerobic fungus. The co-culture of an anaerobic fungus and methanogen has the potential to degrade lignocellulosic substrates to produce methane. More studies are needed in this area to pave the way toward sustainable methane production from lignocellulosic substrates.

## Data Availability Statement

All datasets generated for this study are included in the article/supplementary material.

## Author Contributions

YL and QS completed the experiment and data analysis. YC and ZH conceived and designed the manuscript. YL, YC, and WZ wrote and revised the manuscript.

## Conflict of Interest

The authors declare that the research was conducted in the absence of any commercial or financial relationships that could be construed as a potential conflict of interest.
